# Molecular difference between WASP and N-WASP critical for chemotaxis of T-cells towards SDF-1α

**DOI:** 10.1038/srep15031

**Published:** 2015-10-14

**Authors:** Neeraj Jain, Thirumaran Thanabalu

**Affiliations:** 1 School of Biological Sciences, Nanyang Technological University, 60 Nanyang Drive, Singapore 637551, Republic of Singapore

## Abstract

Wiskott-Aldrich Syndrome protein (WASP) integrates cell signaling pathways to the actin cytoskeleton, which play a critical role in T-cell activation and migration. Hematopoietic cells express both WASP and neural-WASP (N-WASP) which share similar domain structure, yet WASP deficiency causes Wiskott-Aldrich syndrome, suggesting that N-WASP present in the cells is not able to carry out all the functions of WASP. We have identified a unique internal thirty amino acid region (I30) in WASP, which regulates its function in chemotaxis of Jurkat T-cells. Deletion of the I30 region altered the WASP’s closed conformation and impaired its ability to rescue the chemotactic defect of WASP-deficient (Jurkat^WKD^) T-cells. Expression of N-WASP in Jurkat^WKD^ T-cells using WASP promoter restored the migration velocity without correcting the chemotactic defect. However, insertion of I30 region in N-WASP (N-WASP-I30) enabled N-WASP to rescue the chemotactic defect of Jurkat^WKD^ T-cells. N-WASP-I30-EGFP displayed a punctate localization in contrast to the predominant nuclear localization of N-WASP-EGFP. Thus, our study has demonstrated that the I30 region of WASP is critical for localization and chemotaxis. This suggests that N-WASP’s failure to compensate for WASP in rescuing chemotaxis could be due to the absence of this I30 region.

Wiskott Aldrich Syndrome protein (WASP), Neural-WASP (N-WASP) and WASP family Verproline- homologue protein (WAVE) 1, 2, 3 are scaffold proteins that link cell surface signals to actin cytoskeleton^1^,^2^. WASP expression is restricted to the non-erythroid hematopoietic cells while N-WASP and WAVE members are expressed ubiquitously^1^,^3^. WASP deficiency in hematopoietic system leads to Wiskott Aldrich Syndrome (WAS) characterized by thrombocytopenia, eczema, and immunodeficiency^4^. WASP knockout mice showed defects in T-cell activation, decreased peripheral blood lymphocytes and platelet numbers^5^, whereas N-WASP knockout mice are embryonically lethal^6^. In addition, conditional knocking out of N-WASP expression in mouse brain leads to severe hydrocephalus and post-natal death^7^.

WASP and N-WASP share similar protein domain organization, which comprises the WASP homology domain (WH1) at their N-terminus followed by a basic region (BR), GTPase binding domain (GBD), a proline rich region (PRR), and a verproline-cofilin-acidic region (VCA) at C-terminus^1^. The VCA region of WASP family proteins interacts with G-actin and promotes actin polymerization by activating the Arp2/3 complex^8^,^9^. N-WASP has two V-domains, thus, the C-terminal domain (VVCA) can interact with two actin monomers, resulting in superior actin polymerization activity of N-WASP compared to WASP^1^,^10^. Thus WASP and N-WASP share more than 50% sequence homology, having similar binding partners and comparable basic functions.

In resting cells, both WASP and N-WASP exist in an auto-inhibitory closed conformation which prevents the interaction between VCA regions with Arp2/3 complex^11^. Binding of activated Cdc42 with GBD activates WASP and N-WASP by relieving them from their auto-inhibition state, whereas activated Rac1 (member of Rho family of GTPase) activates WAVE 1–3^12^,^13^,^14^. It has also been shown that Rac1 is a more effective activator of N-WASP, while Cdc42 is a more potent activator of WASP^15^. Previously it has been reported that Phosphatidylinositol 4, 5-bisphosphate (PIP2) synergizes with the Cdc42 and mediates WASP and N-WASP activation^16^,^17^. However, Tomasevic *et al.* demonstrated that PIP2 negatively regulates WASP, but not N-WASP activation mediated by Cdc42^15^.

Motility of *Shigella flexneri* in N-WASP-deficient mouse embryonic fibroblast cannot be rescued by the expression of WASP^6^. Nonetheless, WASP and N-WASP can compensate for each other in the motility of Vaccinia virus, and *Mycobacterium marinum*^18^. WASP plays a unique role in osteoclasts sealing zone formation which cannot be compensated by the expression of N-WASP in the WASP-deficient osteoclasts^19^. WASP-deficient macrophages were defective in chemotaxis and unable to form podosomes on their ventral surface^20^,^21^. Moreover, calpain-mediated WASP degradation was found to be crucial since the inhibition of calpain by peptide inhibitors has been shown to reduce podosome turnover rate, resulting in impaired motility of dendritic cells^22^. Compared to WASP, N-WASP is insensitive to calpain^23^. N-WASP expression in WASP-/- hematopoietic stem cells partially restored T-cell function^24^. Normal T-cell development was observed in WASP deficient mice in contrast to impaired T-cell development in WASP- and N-WASP-double knockout mice (DKO) suggesting that WASP and N-WASP play overlapping roles in the development of T-cells^25^,^26^. It has been reported that N-WASP can compensate for WASP-deficiency in macrophage functions such as podosome formation and matrix degradation. However, both WASP and N-WASP are required for Fcγ-R mediated phagocytosis^20^,^27^. These studies suggest that WASP and N-WASP could have both unique and overlapping roles but the molecular mechanism, which can be attributed to these differences, have not been identified.

In order to characterize the molecular differences between WASP and N-WASP, we expressed both of the proteins under WASP promoter in Jurkat^WKD^ T-cells. Expression of WASP but not N-WASP rescued the chemotactic defect of Jurkat^WKD^ T-cells even though both proteins were expressed at comparable levels. WASP has a unique internal thirty amino acid peptide, I30, between the WH1 and BR. WASP I30 deletion mutant, WASP^ΔI30^, adopted a partial open conformation and was not able to rescue the chemotactic defect of Jurkat^WKD^ T-cells. Similarly N-WASP did not rescue the chemotactic defect of Jurkat^WKD^ T-cells. Insertion of the I30 in N-WASP allowed the chimera, N-WASP-I30 to rescue the chemotactic defect of Jurkat^WKD^ T-cells suggesting that this I30 region could be one of the molecular motifs which confers unique activities to WASP.

## Results

### Expression of N-WASP does not rescue chemotactic defects of Jurkat^WKD^ T-cells

**Figure 1:**
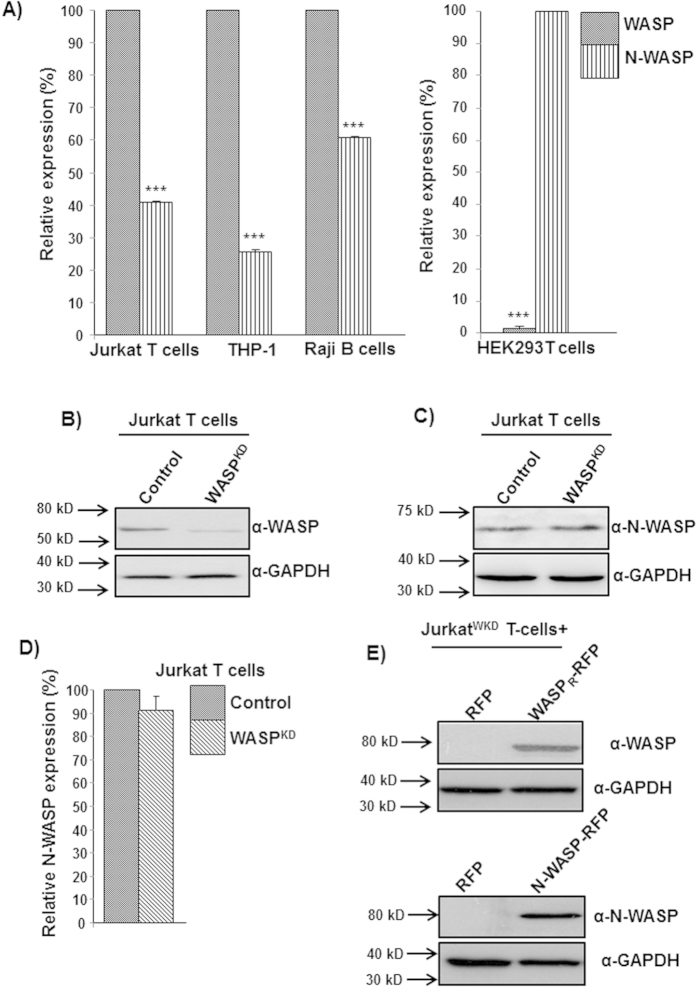
Expression of N-WASP is lower than that of WASP in hematopoietic cells. (**A**) qrtPCR analysis for WASP and N-WASP expression in Jurkat, THP-1 and Raji cells. HEK293T cells were used as negative control for analysis of WASP expression. ***P *<* *0.001* (**B**) Knockdown of endogenous WASP expression in Jurkat T-cells using WASP specific S1-WASP shRNA. (**C**) Expression of N-WASP in wild type and Jurkat^WKD^ T-cells. (**D**) mRNA level of N-WASP quantified by qrtPCR in wild type and Jurkat^WKD^ T-cells. (**E**) Exogenous expression of WASP and N-WASP in Jurkat^WKD^ T-cells.

**Figure 2:**
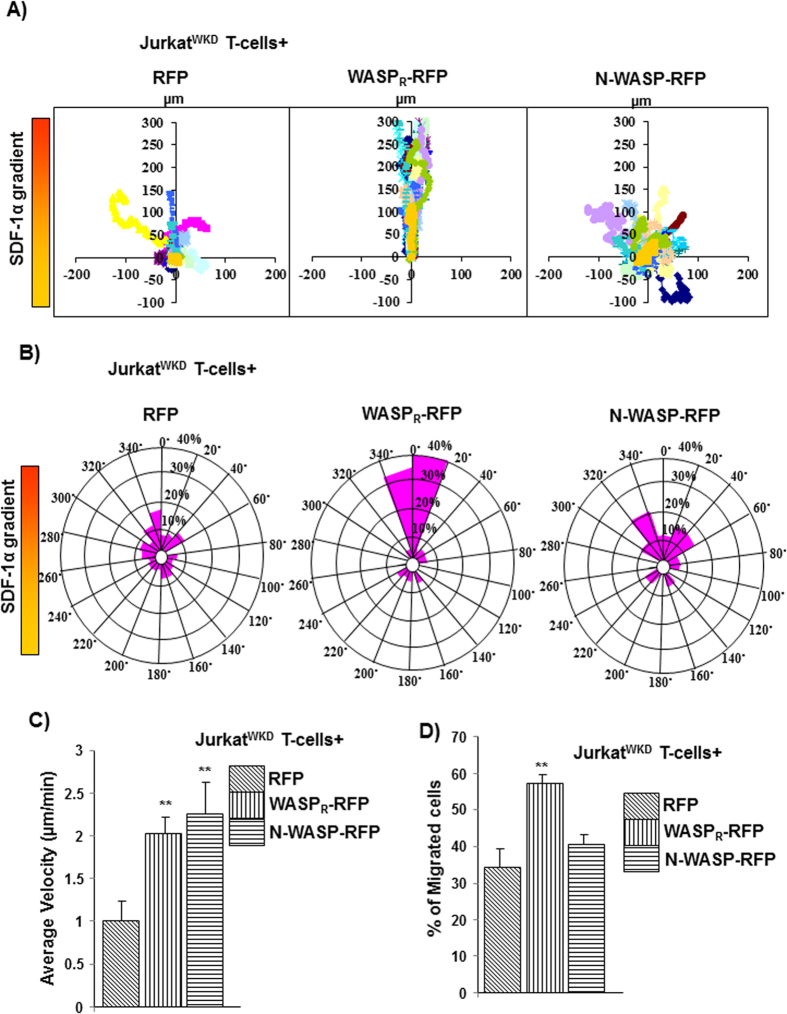
N-WASP expression does not rescue the WASP deficiency in Jurkat T-cells chemotaxis. **(A)** Vector plots representing migration path of 20 randomly selected Jurkat^WKD^ T-cells expressing (1) RFP, (2) WASP_R_-RFP, (3) N-WASP-RFP in Dunn chamber assay exposed to a gradient of chemokine SDF-1α (maximum at top). The intersection point of X and Y axis was taken as starting point of each cell. (**B**) Overall directionality of migration (final position of cell in each 20° sector). (**C**) Migration velocity of total 60 randomly selected cells of cell type as in panel A. **P < *0.01* compared to RFP expressing Jurkat^WKD^ T-cells. (**D**) Transwell migration of Jurkat^WKD^ T-cells expressing (1) RFP, (2) WASP_R_-RFP, (3) N-WASP-RFP represent as percentage of cells migrated. **P < *0.01* compared to RFP expressing Jurkat^WKD^ T-cells.

Expression of WASP has been reported to be 15-fold higher than that of N-WASP in mouse macrophage cell line RAW264.7 using an antibody, which cross-reacts with both WASP and N-WASP^20^. There are no suitable antibodies to detect both WASP and N-WASP with equal affinity, thus we analyzed the expression of WASP and N-WASP in human cell lines (Jurkat, Raji, THP-1) by real-time PCR using specific PCR primers (described in materials and methods). The mRNA level of WASP in these hematopoietic cell lines was ~50% higher than that of N-WASP mRNA and negligible WASP mRNA was observed in control cell line HEK293T cells (Fig. 1A). Similarly, we also found N-WASP mRNA levels to be lower than those of WASP mRNA levels in mouse splenocyte (Fig. S1). Exogenous expression of N-WASP in WASP-deficient macrophages was able to correct defects in chemotaxis and podosome formation^20^. In order to characterize the ability of N-WASP to compensate for WASP deficiency in T-cell functions, WASP-deficient Jurkat T-cell line (WASP^KD^) was generated using S1-WASP shRNA, as described previously^28^ (Fig. 1B). The S1-WASP shRNA used in this study did not target N-WASP (Fig. S2). Knocking down WASP expression in Jurkat T-cells did not affect endogenous N-WASP expression (Fig. 1C,D). Jurkat^WKD^ T-cells were defective in chemotaxis towards chemokine SDF-1α and had reduced migration velocity^29^. Stable exogenous expression of RFP-tagged and shRNA resistant WASP (WASP_R_-RFP) using WASP promoter (Fig. 1E) in Jurkat^WKD^ T-cells rescued the chemotactic defect (Fig. 2). However, exogenous expression of N-WASP-RFP using WASP promoter (Fig. 1E) was unable to rescue the chemotactic defect of Jurkat^WKD^ T-cells in Dunn chemotaxis assay (Fig. 2). Based on circular histogram, the directionality of migration of N-WASP-RFP expressing cells was found to be random. Compared to 71.6% of Jurkat^WKD^ T-cells(WASP_R_-RFP), only 25% of Jurkat^WKD^ T-cells(N-WASP-RFP) were found in the 40° arc facing the chemokine source, which was similar to control (21.6%) (Fig. 2B). Quantitative analysis revealed that N-WASP-RFP expression rescued the migration velocity defect of Jurkat^WKD^ T-cells (2.25 μm/min for Jurkat^WKD^ T-cells(N-WASP-RFP) compared to 1.05 μm/min for Jurkat^WKD^ T-cells(RFP)) and was similar to Jurkat^WKD^ T-cells(WASP_R_-RFP) (1.90 μm/min) (Fig. 2C). The inability of N-WASP-RFP to rescue the chemotactic defect of Jurkat^WKD^ T-cells was further confirmed by transwell migration assay. We found that 40.7% of Jurkat^WKD^ T-cells(N-WASP-RFP) migrated to the lower well compared to 57.10% of Jurkat^WKD^ T-cells(WASP_R_-RFP), suggesting that N-WASP is unable to rescue the chemotaxis defect of Jurkat^WKD^ T-cells towards SDF-1α (Fig. 2D). Taken together, these results demonstrate that N-WASP can rescue the migration velocity but it does not rescue the directional migration defect of Jurkat^WKD^ T-cells.

### A unique internal thirty amino acid region (I30) regulates WASP conformation

**Figure 3:**
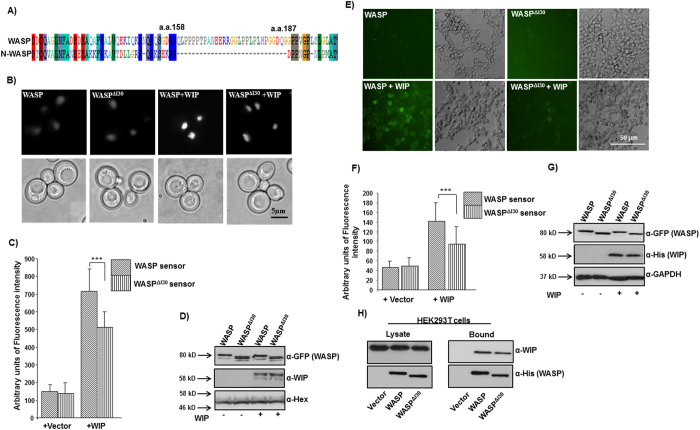
A unique 30 amino acid region of WASP regulates WASP conformation. (**A**) WASP possess proline-rich 30 amino acid region (a.a.158–187) located between WASP homology domain 1 (WH1) and basic region (BR). (**B**) *S. cerevisiae* cells were transformed with empty vector or NLS-WIP together with (1) WASP sensor or (2) WASP^ΔI30^ sensor. The cells were grown to exponential phase and YFP signals were analyzed. Bar = 5 μm. (**C**) Quantification of fluorescence signal from 100 *S. cerevisiae* cells expressing plasmids as described in panel B. ***P < *0.001*. (**D**) Analysis of expression of the WASP sensors or WIP in *S. cerevisiae* cells. Anti-Hexokinase (α-Hex) was used for endogenous control. (**E**) HEK293T cells expressing empty vector or WIP together with sensor constructs (WASP or WASP^ΔI30^) analyzed by fluorescence microscopy. (**F**) Quantification of fluorescence signal from HEK293T cells expressing plasmid as described in panel E. ***P < *0.001*. (**G**) Expression of sensors constructs (WASP or WASP^ΔI30^) or WIP in HEK293T cells. (**H**) HEK293T cells were transfected with WIP together with (1) vector (2) WASP-His (3) WASP^ΔI30^-His. The WASP or WASP deletion mutants were isolated by His-tag pull-down assay and probed for the presence of WIP.

Sequence alignment of WASP and N-WASP revealed the presence of a unique internal thirty amino acid region (a.a. 158–187) in WASP protein between WH1 domain and BR (Fig. 3A). In this study, this thirty amino acid region of WASP was named the I30 region of WASP. The I30 region of WASP contains nine proline residues out of thirty amino acid (30% Proline), suggesting that beside the proline-rich region of WASP (PRR: a.a. 312–417), the I30 region could also mediate interaction with SH3 domain-containing proteins. In order to test the functional importance of I30 region, we first deleted this I30 region from WASP (WASP^ΔI30^). We have previously reported that expression of human WASP with human WIP could rescue the growth defect of *S. cerevisiae las17*Δ strain and growth rescue required a functional VCA domain as well as WASP-WIP interaction^30^. Expression of human WASP^ΔI30^ with human WIP rescued the growth defect of *S. cerevisiae las17*Δ strain (Fig. S3) suggesting that WASP^ΔI30^ can form functional complex with WIP and that the VCA domain in WASP^ΔI30^ plays an important role in the rescue of growth defect. WASP has been shown to form a closed conformation in the resting state, which is stabilized by WIP. In order to analyze the conformation of WASP^ΔI30^, we performed a Bi-molecular Fluorescence Complementation (BiFC) assay as described previously^28^,^31^. The YFP molecule was split in to two fragments and fused to the termini of WASP or WASP^ΔI30^ (NLS-YFP_1-154_-WASP-YFP_155-238_ or NLS-YFP_1-154_-WASP^ΔI30^-YFP_155-238_) to form a sensor molecule. To protect WASP sensor from cytoplasmic proteases, NLS was attached to the N-termini of YFP_1-154_. The fluorescence intensity of both WASP and WASP^ΔI30^ sensor was increased in the presence of WIP suggesting that WIP binds to the WH1 domain of WASP and stabilizes the closed conformation. However the fluorescence intensity of WASP^ΔI30^ sensor was reduced compared to WASP sensor in the presence of WIP (Fig. 3B,C). The reduction in fluorescence intensity of WASP^ΔI30^ sensor was neither due to its poor expression (Fig. 3D) nor due to loss of its interaction with WIP (Fig. 3H). A similar result was obtained in mammalian system when WASP or WASP^ΔI30^ sensors were expressed in HEK293T cells together with WIP (Fig. 3E–G). We have previously shown that Toca1 and Nck1 can relieve WASP from its closed conformation even in the presence of WIP^31^. In this study, we found that Nck1 and Toca1 can relieve the auto-inhibitory state of WASP sensor in the presence of WIP but have no effect on the conformation of WASP^ΔI30^ sensor (Fig. S4). Our data therefore suggests that deletion of I30 region could cause WASP to adopt a partially open conformation even in the presence of WIP.

### WASP^ΔI30^ expression does not rescue the chemotactic defect of Jurkat^WKD^ T-cells towards SDF-1α

**Figure 4:**
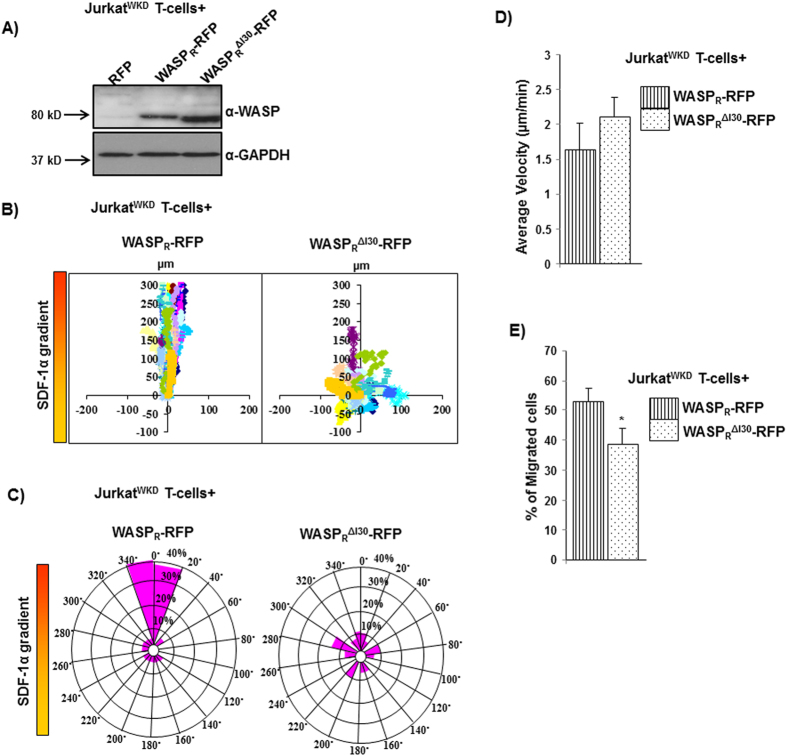
I30 region of WASP critical for Jurkat T-cell chemotaxis. (**A**) Expression levels of RFP tagged WASP_R_ and WASP_R_^ΔI30^ in Jurkat^WKD^ T-cells. (**B**) Vector plots representing the migration path of Jurkat^WKD^ T-cells expressing (1) WASP_R_-RFP, (2) WASP_R_^ΔI30^-RFP in Dunn chamber assay. (**C**) Circular histograms for analysis of overall directionality of migration (final position of cells in each 20° sector). (**D**) Migration velocity of total 60 randomly selected cells of cell type as in panel B. (**E**) Transwell migration of Jurkat^WKD^ T-cells expressing (1) WASP_R_-RFP, (2) WASP_R_^ΔI30^-RFP and percentage of cells migrated to the bottom well was counted. *P < *0.05*.

In order to analyze the role of the I30 region of WASP in T-cell function, we expressed RFP-tagged WASP constructs (WASP_R_ or WASP_R_^ΔI30^) in Jurkat^WKD^ T-cells and analyzed their chemotactic response towards SDF-1α using a Dunn chamber (Fig. 4A). Expression of WASP_R_, but not WASP_R_^ΔI30^, rescued the chemotactic defect of Jurkat^WKD^ T-cells (Fig. 4B–E). Quantitative analyses of migration velocity revealed that the expression of either WASP_R_ (1.63 μm/min) or WASP_R_^ΔI30^ (2.1 μm/min) restored migration velocity of Jurkat^WKD^ T-cells (Fig. 4D). We observed that WASP_R_^ΔI30^ expressing Jurkat^WKD^ T-cells frequently changed their migration direction compared to WASP_R_ expressing cells (data not shown). Circular histograms showed that only 22.32% of Jurkat^WKD^ T-cells(WASP_R_^ΔI30^), as compared to 78.3% of Jurkat^WKD^ T-cells(WASP_R_),were found within the 40° arc facing the chemokine source, suggesting that the migration of WASP_R_^ΔI30^ expressing cells was random (Fig. 4C). The migration defect of Jurkat^WKD^ T-cells expressing WASP_R_^ΔI30^ was further confirmed by transwell migration assay. The migration of WASP_R_^ΔI30^ expressing Jurkat^WKD^ T-cells towards SDF-1α in transwell migration assay was significantly impaired, with 38.88% of total Jurkat^WKD^ T-cells(WASP_R_^ΔI30^) migrated to the lower well compared to 52.90% of total Jurkat^WKD^ T-cells(WASP_R_) (Fig. 4E). Taken together, these results suggest that the I30 region of WASP could play an essential role in establishing or maintaining the directionality of Jurkat T-cells movement towards chemokine SDF-1α.

### N-WASP-I30 localizes to punctate structures at cell cortex

**Figure 5:**
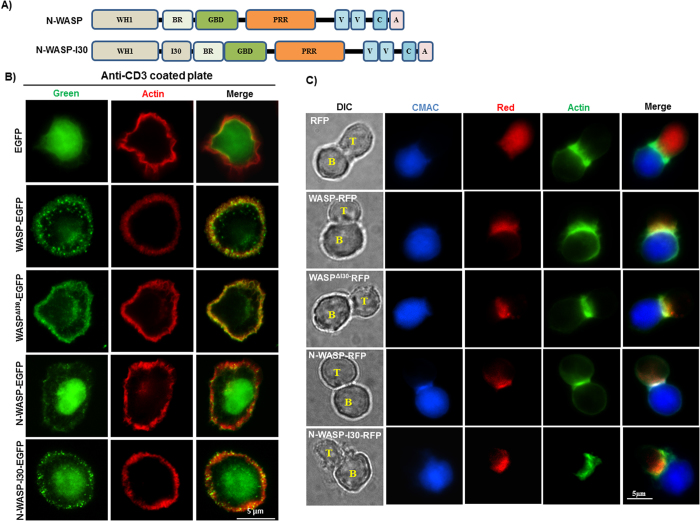
I30 region of WASP recruit N-WASP-I30 to punctate structures at cell cortex. (**A**) WASP-I30 region was inserted in N-WASP between WH1 domain and basic region of N-WASP. (**B**) Jurkat T-cells expressing EGFP tagged WASP_R_, WASP_R_^ΔI30^, N-WASP and N-WASP-I30 were stimulated on anti-CD3 (OKT3)-coated coverslip for 5 min and stained with Alexa fluor 594 phalloidin. The experiment has been repeated three times and 25 cells were analyzed each time. (**C**) Jurkat T-cells expressing WASP_R_-RFP, WASP_R_^ΔI30^-RFP, N-WASP-RFP and N-WASP-I30-RFP were allowed to form conjugate with Raji B cell initially pulsed with SEE toxin. The conjugates between T: B cells were detected by Alexa fluor 488 phalloidin. Bar = 5 μm.

Expression of N-WASP did not rescue the chemotaxis of Jurkat^WKD^ T-cells (Fig. 2); furthermore, deletion of I30 region of WASP abolished WASP function in rescuing the chemotaxis of Jurkat^WKD^ T-cells (Fig. 4), suggesting that the I30 region of WASP could be critical for chemotaxis. In order to verify the importance of the I30 in chemotaxis, we inserted the I30 region of WASP in N-WASP between the WH1 domain and BR to obtain N-WASP-I30 (Fig. 5A). Both WASP and N-WASP have been shown to be recruited to T and B cell contact site and PRR domain of these proteins is essential for such recruitment^32^. Since I30 region of WASP is 30 % proline rich, we tested whether I30 plays a role in recruitment to TCR activation site by analyzing the localization of EGFP-tagged WASP_R_, WASP_R_^ΔI30^, N-WASP and N-WASP-I30 constructs (expression under the regulation of CMV promoter) in Jurkat T-cells. Jurkat T-cells expressing different constructs were plated on anti-CD3 coated plate, fixed after 5 min of stimulation on anti-CD3 coated surface, and stained with Alexa fluor 594 phalloidin. In the absence of anti-CD3 stimulation, cells did not spread and the localization of WASP was throughout the cells (data not shown). Upon stimulation by anti-CD3 the cells were well spread and WASP was found to localize to peripheral actin ring with punctate localization in 89.34 ± 8.3% of cells. WASP_R_^ΔI30^ was also found in the cortical area of the cell, however the punctuate localization was reduced to 38.67 ± 4.6% of cells compared to WASP_R._ N-WASP was also found to be localized at the actin rich circumferential ring. But compared to WASP, most of the N-WASP was found at the cell center (nucleus) of the spread cell. N-WASP-I30 was also observed in the nucleus, however, with reduced fluorescence intensity and more intense punctate structures at cell periphery in 30.66 ± 2.3 % of cells. This suggests that the I30 region could play a role in localizing WASP to prominent punctate structures at cell periphery (Fig. 5B). We also examined the localization pattern of WASP_R_, WASP_R_^ΔI30^, N-WASP and N-WASP-I30 in Jurkat T-cells conjugated with Raji B cells (pulsed with staphylococcal enterotoxin E (SEE) toxin) and found that there was no difference in the localization among these proteins, all of them localized to T and B cell contact site, suggesting that I30 region of WASP may not be critical for WASP localization at immunological synapse (Fig. 5C).

### N-WASP-I30 rescued the chemotactic defect of Jurkat^WKD^ T-cells

**Figure 6:**
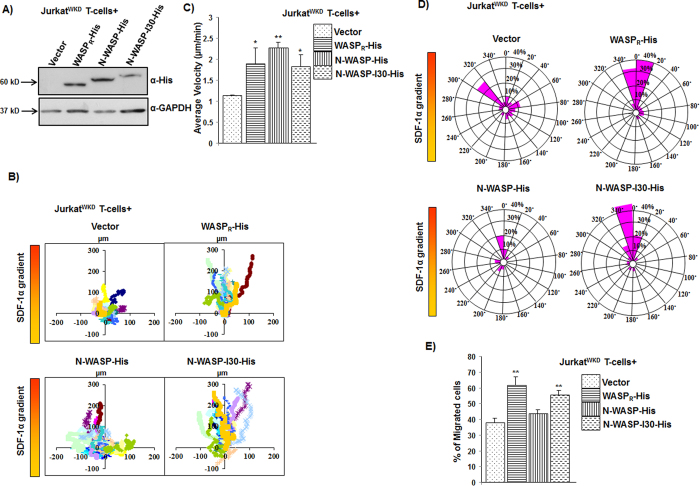
Expression of N-WASP-I30 rescued the defective chemotaxis of WASP^KD^ Jurkat T cells. (**A)** Expression levels of His tagged WASP_R_ and N-WASP, N-WASP-I30 and vector alone in Jurkat^WKD^ T-cells. (**B)** Vector plots representing the migration path of Jurkat^WKD^ T-cells expressing (1) WASP_R_-His, (2) N-WASP-His, (3) N-WASP-I30-His in Dunn chamber assay. (**C)** Migration velocity of total 60 randomly selected cells of cell type as in panel B. *P < *0.05*, **P < *0.01* compared to vector expressing Jurkat^WKD^ T-cells. (**D**) Overall directionality of Jurkat^WKD^ T-cells expressing plasmid as in panel B. (**E**) Transwell migration of Jurkat^WKD^ T-cells expressing (1) WASP_R_-His, (2) N-WASP-His, (3) N-WASP-I30-His. **P < *0.01* compared to vector expressing Jurkat^WKD^ T-cells.

In order to examine the expression pattern of WASP, N-WASP and N-WASP-I30 in Jurkat T-cells, the corresponding sequences were tagged with His-tag (6 X Histidine) and cloned into neomycin resistant plasmid. The resulting WASP_R_-His, N-WASP-His, N-WASP-I30-His and vector alone were microporated into Jurkat^WKD^ T-cells and subsequently selected with neomycin for one week (Fig. 6A). The chemotactic responses of neomycin-selected cells (stable cells) towards SDF-1α were analyzed using Dunn chamber. WASP_R_-His rescued the chemotactic defect of Jurkat^WKD^ T-cells, whereas N-WASP-His expressing cells had impaired migration towards the chemokine gradient (Fig. 6B). However, expression of N-WASP-I30-His rescued the chemotactic defect of Jurkat^WKD^ T-cells. The migration velocities of Jurkat^WKD^ T-cells(N-WASP-His) at 2.26 μm/min, or Jurkat^WKD^ T-cells(N-WASP-I30-His) at 1.82 μm/min were comparable to Jurkat^WKD^ T-cells(WASP_R_-His) at 1.89 μm/min (Fig. 6C). Circular histogram of the final position of the cells showed that 66.6% of total Jurkat^WKD^ T-cells(N-WASP-I30-His) lie within the 40° arc facing the chemokine source, which was comparable to the 73.3% recorded for Jurkat^WKD^ T-cells(WASP_R_-His), whereas only 30% of Jurkat^WKD^ T-cells(N-WASP-His) were found within the 40° arc facing the chemokine source (Fig. 6D). The ability of N-WASP-I30-His to rescue the migration defects of Jurkat^WKD^ T-cells was confirmed by transwell migration assay. Similar to chemotactic defect observed in Dunn chamber, the migration of Jurkat^WKD^ T-cells(N-WASP-His) was significantly impaired in transwell migration assay, 43.5% of total Jurkat^WKD^ T-cells(N-WASP-His) and 37.6% of total Jurkat^WKD^ T-cells(Vect) migrated to the lower well containing SDF-1α compared to 61.4% total of Jurkat^WKD^ T-cells(WASP_R_-His). However 55.48% of Jurkat^WKD^ T-cells(N-WASP-I30-His) migrated towards the chemokine source which was comparable to WASP_R_-His expressing cells (Fig. 6E). Taken together, these results suggest that I30 region of WASP could be important for directional migration of Jurkat T-cells towards the chemokine (SDF-1α) source. Insertion of I30 region of WASP in N-WASP enabled it to mediate Jurkat T-cells chemotaxis towards SDF-1α.

## Discussion

Hematopoietic cells express both WASP and N-WASP, which are required for T-cell development^25^. WASP deficiency causes Wiskott Aldrich syndrome suggesting that N-WASP present in these cells is not able to perform all the functions of WASP^20^. A number of studies using N-WASP to rescue the defects of WASP-deficient cells have suggested that both of these two proteins have unique as well as redundant roles in hematopoietic^20^,^25^ and other cells^18^,^19^. However, the molecular differences in the proteins giving rise to the unique roles of WASP in T-cell functions have not been characterized.

We have found that the N-WASP mRNA levels was 40–66% lower than that of WASP mRNA (Fig. 1A) in hematopoietic cell lines consistent with the previous studies in which N-WASP expression was found to be lower than that of WASP expression in the cells of myeloid lineages^20^,^23^. We also found that mRNA levels of N-WASP are lower than that of WASP mRNA levels in mouse splenocyte in resting (18.7-fold lower) or activated state (40-fold lower; when activated with anti-CD3 or 32-fold lower when activated by anti-CD3/CD28 antibodies) (Fig. S1). Unlike N-WASP, WASP is regulated by proteolytic degradation^23^,^33^ and the higher levels of WASP mRNA detected could be a compensatory mechanism to produce sufficient WASP protein. T cells from WAS patients are defective in homing, chemotaxis towards chemokine SDF-1α and IL-2 production in response to TCR activation^34^,^35^. Consistent with these studies, knocking down of WASP expression in Jurkat T-cells impaired the chemotaxis of Jurkat^WKD^ T-cells towards chemokine SDF-1α in Dunn chamber and transwell migration assay (Fig. 2). Expression of either WASP or N-WASP under WASP promoter in Jurkat^WKD^ T-cells rescued the IL-2 production defect of Jurkat^WKD^ T-cells (Data not shown) and thus was not investigated any further. The chemotactic defect of Jurkat^WKD^ T-cells was rescued by exogenous WASP expression, but it was only partially rescued by N-WASP expression (Fig. 2). Although the migration velocity of Jurkat^WKD^ T-cells(N-WASP) was comparable to Jurkat^WKD^ T-cells(WASP_R_), the migration was random. It has been reported that over-expression of N-WASP in WASP-deficient macrophages cell line restored podosome-dependent matrix degradation and chemotaxis^20^ and it was suggested that the inability of N-WASP to completely compensate for the loss of WASP could be due to insufficient expression of N-WASP. However, in our experimental system N-WASP was not able to correct the chemotactic defect of Jurkat^WKD^ T-cells even when both WASP and N-WASP were expressed at comparable levels under the transcriptional regulation of WASP promoter (Fig. 6). WASP-deficient macrophages can form directional protrusions towards the chemokine (CSF-1) source, but the persistency of directional protrusions was reduced, resulting in random migration of macrophage cells^36^. Thus, it is possible that the inability of N-WASP in restoring chemotactic defect of Jurkat^WKD^ T-cells could be due to the defective polarization of Jurkat^WKD^ T-cells (N-WASP) or altered persistency of directional protrusions (Fig. S6).

WASP has a unique internal thirty amino acid region, WASP_158-187_ (between WH1 and BR domain) which we named as I30 (Fig. 3A). Expression of WASP^ΔI30^ did not rescue the chemotactic defect of Jurkat^WKD^ T-cells as determined by Dunn chamber assay and transwell migration assay (Fig. 4). More significantly, Jurkat^WKD^ T-cells(WASP^ΔI30^) behaved similar to Jurkat^WKD^ T-cells(N-WASP) cells in terms of their chemotactic response to SDF-1α (defective directionality of migration without affecting migration velocity). Deletion of I30 region of WASP did not affect WASP-WIP interaction (mediated by the WH1 domain of WASP) (Fig. 3H) and function of VCA domain of WASP as determined by growth rescue experiment of *las17*Δ yeast strain (Fig. S3), suggesting that deletion of I30 region did not lead to any global change in the conformation of WASP. The I30 region of WASP contains 9 proline residues out of 30 amino acid (30% Proline), indicating that the I30 region may also mediate interaction with SH3 domain-containing proteins similar to the proline-rich region of WASP. We carried out a Yeast two-hybrid screen with two human cDNA libraries using WASP_138-265_ as a bait but could not identify any novel WASP binding proteins. Thus we carried out a yeast two-hybrid analysis using WASP_138-320_ or WASP_138-320_^ΔI30^ with known WASP PRR binding proteins (Fyn, Fgr, Lyn, Hck, Nck1, Toca1, Grb2, Src, PIK, Tec, VASP, Lck, PSTPIP, Btk, Abl, IRSp53, Cortactin). We found that WASP_138-320_ can interact with Hck, Fyn, Nck, Grb2, Fgr, Lyn, Toca1, Src but WASP_138-320_^ΔI30^ could not suggesting that I30 may mediate interaction with these 8 proteins (data not shown). Interaction of Hck with I30 region of WASP was further confirmed by His-tag pull-down assay (Fig. S5). Deletion of the I30 region of WASP partially opened the WASP closed conformation, even in the presence of WIP (Fig. 3), and caused an increase in the Hck-mediated WASP-Tyr^291^ phosphorylation (Fig. S5). This is probably due to the increased accessibility of Tyr^291^ residue of WASP to the tyrosine kinase, consistent with the previous finding, which showed that the activating mutation of WASP^L270P^ (identified in patients with severe congenital neutropenia) stabilizes WASP in an open conformation^37^, and increases Tyr^291^ phosphorylation of WASP^27^,^38^. The tyrosine phosphorylation of WASP was shown to be required for the directional response of macrophage cells towards CSF-1, thus it plays a critical role in coordinating actin cytoskeleton rearrangement, which is essential for maintaining persistency of directional migratory protrusions^36^. Podosomes in macrophages from XLN patients (who have a point mutation I294T in the GTPase-binding domain of WASP) are highly dynamic in nature with high rate of turnover^39^ resulting in defective migration. This is due to an open conformation of WASP^I294T^ mutant that increases WASP-Tyr^291^ phosphorylation and promotes WASP^I294T^ degradation, resulting in the increased podosome disassembly^39^. Our observations imply that a right balance between WASP activation and degradation is necessary for the regulating actin polymerization, which is vital for directional migration.

In order to determine the fundamental role of WASP I30 region in chemotaxis, we generated N-WASP-I30, by inserting WASP I30 peptide in N-WASP between the WH1 domain and BR (Fig. 4A). Both WASP^ΔI30^ and N-WASP-I30 was able to localize at the immunological synapse, suggesting that the I30 region of WASP is not critical for localization at TCR activation site. This is consistent with previous studies, which have established that the PRR region of WASP is sufficient for localization at TCR activation sites^29^,^32^.

Expression of N-WASP-I30 rescued the chemotactic defect of Jurkat^WKD^ T-cells towards SDF-1α, as determined by Dunn chamber assay and transwell assay (Fig. 6). This suggests that the I30 region of WASP could be required either to mediate interactions with proteins and/or alter the conformation of N-WASP necessary for regulating chemotaxis of T-cells. WASP is sensitive to calpain, while N-WASP is resistant to calpain proteases^23^, and the inhibition of calpain has been shown to stabilize podosomes, resulting in impaired migration of DCs, suggesting that calpain-mediated turnover of WASP is essential for chemotaxis^22^. We found that the expression of N-WASP-I30 was slightly reduced compared to N-WASP (Fig. 6A). However, this is not due to any change in the sensitivity of N-WASP-I30 to calpain (data not shown). The slight reduction in N-WASP-I30 expression in Jurkat T-cells could be due to other proteolytic pathways.

We have shown that the unique thirty amino acid peptide, I30, is critical for WASP-regulated chemotaxis. Insertion of this I30 peptide into N-WASP conferred N-WASP-I30 the ability to compensate for WASP deficiency in T-cell chemotaxis. Our study suggests that the proline-rich I30 peptide could mediate interaction with proteins critical for chemotaxis or promote WASP to adopt a conformation necessary for chemotaxis.

## Materials and Method

### Cell culture, transfection and stable cell generation

HEK293T cells and Phoenix Amphotropic packaging cell line (ATCC, USA) were maintained in DMEM medium supplemented with 10% FBS, while Jurkat T-cells (clone E6-1; ATCC, USA) were maintained in RPMI-1640 medium with 10% FBS at 37 °C incubator. Jurkat^WKD^ T-cells were generated as described previously^28^,^29^. In brief, Amphotropic cells were used to generate retrovirus expressing human WASP specific shRNA (S1-WASP-shRNA), which was used to transduce Jurkat T-cells. Jurkat T-cells stably expressing GFP (transduced cells) were FACS sorted and Western blot analysis was used to analyze endogenous WASP expression and knockdown efficiency. In order to reconstitute the WASP expression in Jurkat^WKD^ T-cells, four silent point mutations were made in WASP gene targeted by S1-WASP-shRNA. WASP_R_ (shRNA resistant WASP) or deletion construct or N-WASP was tagged with RFP or His tag were microporated (Neon transfection system; Invitrogen, CA, USA) in Jurkat^WKD^ T-cells as described^28^. Transfected cells were selected with neomycin (1.5 mg/ml) (G418, P02–012; PAA Laboratories, Pasching, Austria) for one week and expression of exogenous gene was confirmed with Western blot using appropriate antibodies. Mouse monoclonal anti-WASP (D1), and anti-GAPDH antibodies were purchased from Santa Cruz and Ambion, respectively. Anti-His antibody was purchased from Delta Biolabs. All secondary antibodies conjugated with horseradish peroxidase were obtained from Sigma-Aldrich (St. Louis, MO, USA).

### Quantitative real time PCR

Total RNA was isolated from THP-1 cells, HEK293T cells, Jurkat T-cells and Raji B-cells. RNA was converted into cDNA and quantitative real time PCR was performed for WASP and N-WASP using thermal cycler (Applied Biosystem 7500) and SYBR Green/ROX qPCR Master Mix (Fermentas). The sense and anti-sense primer for human WASP were 5′-CTGTGTGCTTCGTGAAGGATAA and 5′-TCGTCTGCAAAGTTCAGCCC; human N-WASP were 5′-AAGGATGGGAAACTATTGTGGGA and 5′-GACGGCCCAAAAGGTCTGTAA; MRPL-27 primers 5′-CTGGTGGCTGGAATTGACCGCTA and 5′-CAAGGGGATATCCACAGAGTACCTTG) were used for normalization.

### Chemotaxis assay

Chemotaxis assay was performed in this study using Dunn chamber (Weber Scientific International, UK)^40^ as described previously^29^. In summary, Jurkat^WKD^ T-cells expressing WASP or N-WASP constructs were first allowed to attach to fibronectin (2 μg/ml) coated coverslip and the coverslip was then inverted on to Dunn chamber filled with complete RPMI media. The media from outer well of Dunn chamber was replaced with RPMI media containing chemokine SDF-1α (5 nM; Peprotech, London, UK). The edges of the coverslip were sealed with mixture of hot wax. Migrating cells on the annular bridge of the Dunn chamber were imaged using time lapse video microscopy (Olympus IX81) fitted with CoolSNAP^HQ^ camera at 2 min interval for period of 3 hours. The images were analyzed and the migration path of cells was traced using Metamorph Software (Molecular Device, CA, USA). Chemotaxis assay was also performed using Transwell (5.0 μm pore size, Costar, Cambridge, MA). The bottom well of transwell was filled with SDF-1α (100 ng/ml) containing media and 2 × 10^5^ cells in 100 μl media were added to transwell. Cells were allowed to migrate for 3 hours and the cells migrated to the bottom well were counted and plotted as percentage of cells migrated.

### Immunofluorescence microscopy and T cell-APC conjugate formation

Jurkat^WKD^ T-cells stably expressing WASP or N-WASP or deletion constructs were seeded on anti-CD3 (clone OKT3; eBioscience) (10 μg/ml) coated coverslip. Cells after 5 min incubation were fixed with 3.7% formaldehyde in PBS and stained with Alexa fluor 594 phalloidin. For conjugate formation, Raji B cells were first stained with CMAC (Molecular probes) for 30 min and then incubated for 90 min with SEE superantigen (2 μg/ml; Toxin Technologies) at 37 °C. Equal number of SEE toxin pulsed Raji B cells mixed with Jurkat T-cells followed by incubation for 30 min at 37 °C. The conjugates were allowed to adhere on poly-l-lysine-coated coverslip for 10 min. Cells were fixed with 3.7% formaldehyde, stained with Alexa fluor 488 phalloidin. Fluorescent images were captured using Olympus microscope fitted with CoolSNAP^HQ2^ camera.

### Bimolecular Fluorescence Complementation Assay (BiFC)

BiFC assay was performed as described previously^31^. In brief, *Saccharomyces cerevisiae* strain PJ69-4A was transformed with WASP or WASP^ΔI30^ sensor plasmid together with empty vector or NLS-WIP. Cells after transformation were plated on selection plate (-Trp-Leu). The transformed yeast cells were grown till their exponential phase in YPUAD (yeast extract, peptone, uracil, adenine and dextrose) media and visualized using fluorescence microscope (Olympus, Roper scientific). Fluorescence intensity was quantified using Metamorph software. Similar experiment was performed in mammalian system using HEK293T cells. HEK293T cells were transfected with WASP or WASP^ΔI30^ sensor plasmid together with empty plasmid or WIP-His. The fluorescence images were captured 36 hours after transfection and fluorescence intensity was quantified. For Western blot analyses using yeast cells, lysis and sample preparation method was followed as described^31^.

### Statistical analysis

Unpaired student’s t-test was performed for statistical significance analysis and *p* value < 0.05 was considered as significance. Values in bar charts represent the mean ± S.D from three independent experiments.

## Additional Information

**How to cite this article**: Jain, N. and Thanabalu, T. Molecular difference between WASP and N-WASP critical for chemotaxis of T-cells towards SDF-1α. *Sci. Rep.* **5**, 15031; doi: 10.1038/srep15031 (2015).

## Supplementary Material

Supplementary Information
