# Supplementary material for: Molecular difference between WASP and N-WASP critical for chemotaxis of T-cells towards SDF-1α
Source: Sci Rep. 2015 Oct 14;5:15031. doi: 10.1038/srep15031 (PMC4604493; doi:10.1038/srep15031)
Supplement: Supplementary Information [file srep15031-s1.pdf]

Molecular difference between WASP and N-WASP critical for chemotaxis of T-cells towards SDF-1α

Neeraj Jain and Thirumaran Thanabalu*

Figure S1: Expression of N-WASP is lower than WASP in primary T cells. Quantitative real time PCR analysis of WASP and N-WASP expression at mRNA level in non-activated and activated splenocytes. *** $P<0.001$

Figure S2: S1 WASP shRNA was specific to WASP. Expression of WASP-GFP, N-WASP-GFP and $WASP_R$-GFP in HEK293T cells transfected with or without S1 WASP shRNA using anti-GFP (α-GFP) antibody.

Figure S3: $WASP^{\Delta I30}$ together with WIP can rescue the growth defect of *las17Δ* strain.

A) Viability at 24 and 37°C of *las17Δ* yeast strain IDY166 transformed with full length WT WASP or $WASP^{\Delta I30}$ expressing plasmids with WIP or empty vector (Vect).

B) Western blot analysis of WASP and $WASP^{\Delta I30}$ expressed with WIP or empty vector (Vect) in *las17Δ* yeast strain.

Figure S4: I30 region is critical for Nck1 and Toca1 induced conformation change

A) Quantification of fluorescence signal from 100 *S. cerevisiae* cells transformed with NLS-WIP + WASP sensor or NLS-WIP + $WASP^{\Delta I30}$ sensor together with (1) Vector or (2) Nck1 (3) Toca1 expressing plasmids.***$P<0.001$ compared to WASP sensor + NLS WIP + Vector. .**$P<0.01$

B) Analysis of expression of the WASP sensors or WIP in *S. cerevisiae* cells. Anti-Hexokinase (α-Hex) was used for endogenous control.

Figure S5: I30 region interacts with Hck.

(A) Schematic diagram showing the WASP and WASP deletion mutants generated for His-tag pull down assay**.**

(B) HEK293T cells were transfected with Hck together with (1) Vector (2) WASP-His, (3) $WASP^{\Delta I30}$-His, (4) $WASP_{138\text{-}320}$-His (5) $WASP_{138\text{-}320}{}^{\Delta I30}$-His. The WASP or WASP deletion mutants were isolated by His-tag pull-down assay. * represent the degradation product of WASP or its deletion mutants.

(C) Deletion of I30 region enhanced Hck mediated WASP phosphorylation. HEK293T cells were transfected with WASP-His or $WASP^{\Delta I30}$-His together with (1) Vector, (2) Hck-GFP. WASP or $WASP^{\Delta I30}$ was isolated by His-tag pull-down assay. Hck expression was detected using anti-GFP antibody. Tyrosine-phosphorylation levels of WASP and $WASP^{\Delta I30}$ was detected using 4G10 antibody.

Figure S6: Directionality of migration is random in N-WASP expressing $Jurkat^{WKD}$ T-cells.

Time lapsed images of $WASP_R$ or N-WASP expressing $Jurkat^{WKD}$ T-cells in Dunn chamber assay.

Supplementary

S1)

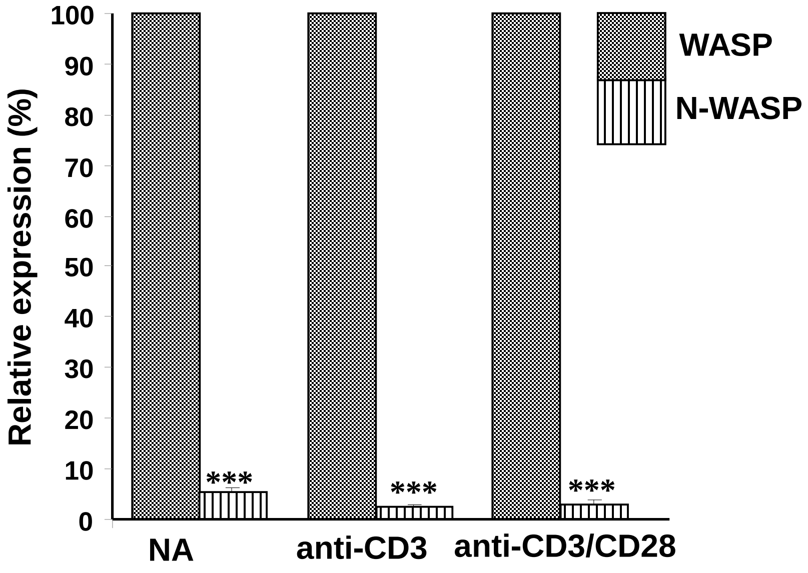

Supplementary

S2)

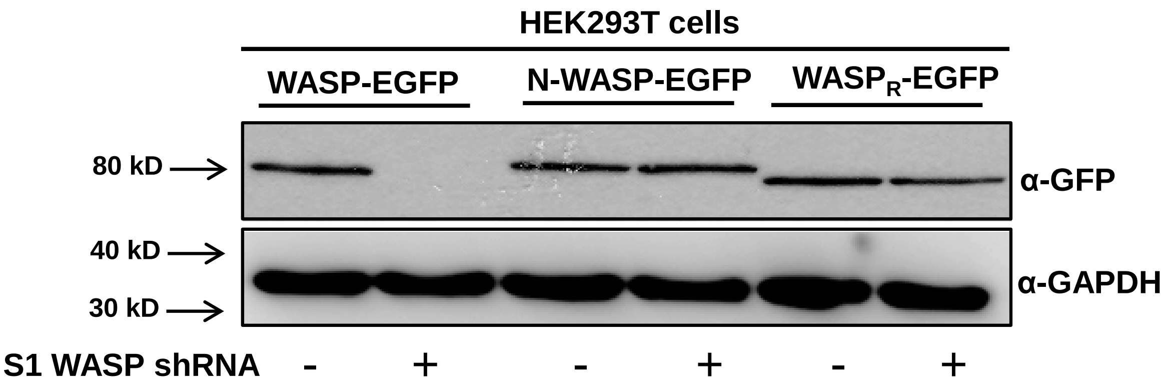

S3)

A)

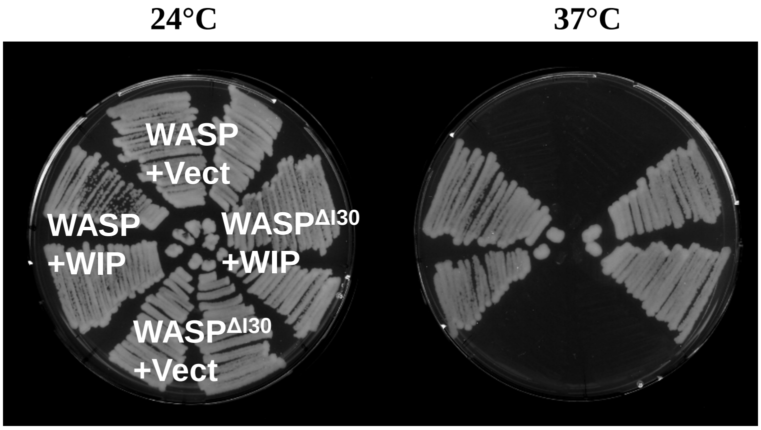
24°C
37°C
WASP +Vect
WASP +WIP
WASP$^{\Delta I30}$ +WIP
WASP$^{\Delta I30}$ +Vect

B)

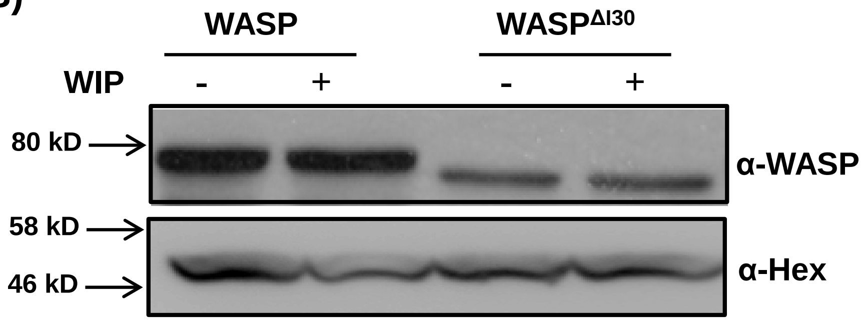
WASP
WASP$^{\Delta I30}$
WIP
-
+
-
+
80 kD
58 kD
46 kD
α-WASP
α-Hex

S4)

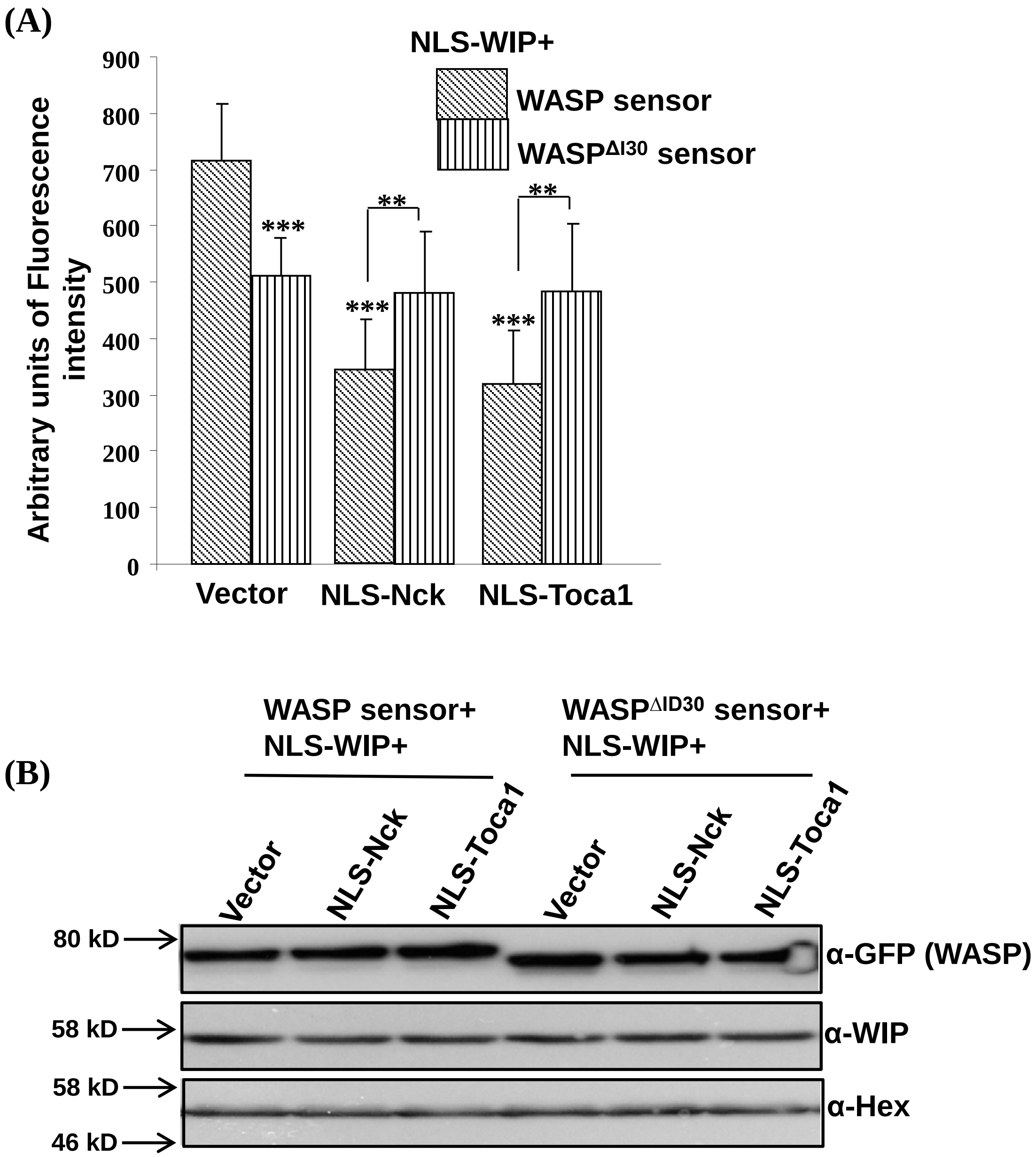
(A)
NLS-WIP+
WASP sensor
WASPΔI30 sensor
Arbitrary units of Fluorescence intensity
900
800
700
600
500
400
300
200
100
0
***
**
***
**
***
Vector
NLS-Nck
NLS-Toca1
(B)
WASP sensor+
NLS-WIP+
WASPΔID30 sensor+
NLS-WIP+
Vector
NLS-Nck
NLS-Toca1
Vector
NLS-Nck
NLS-Toca1
80 kD
α-GFP (WASP)
58 kD
α-WIP
58 kD
46 kD
α-Hex

S5)

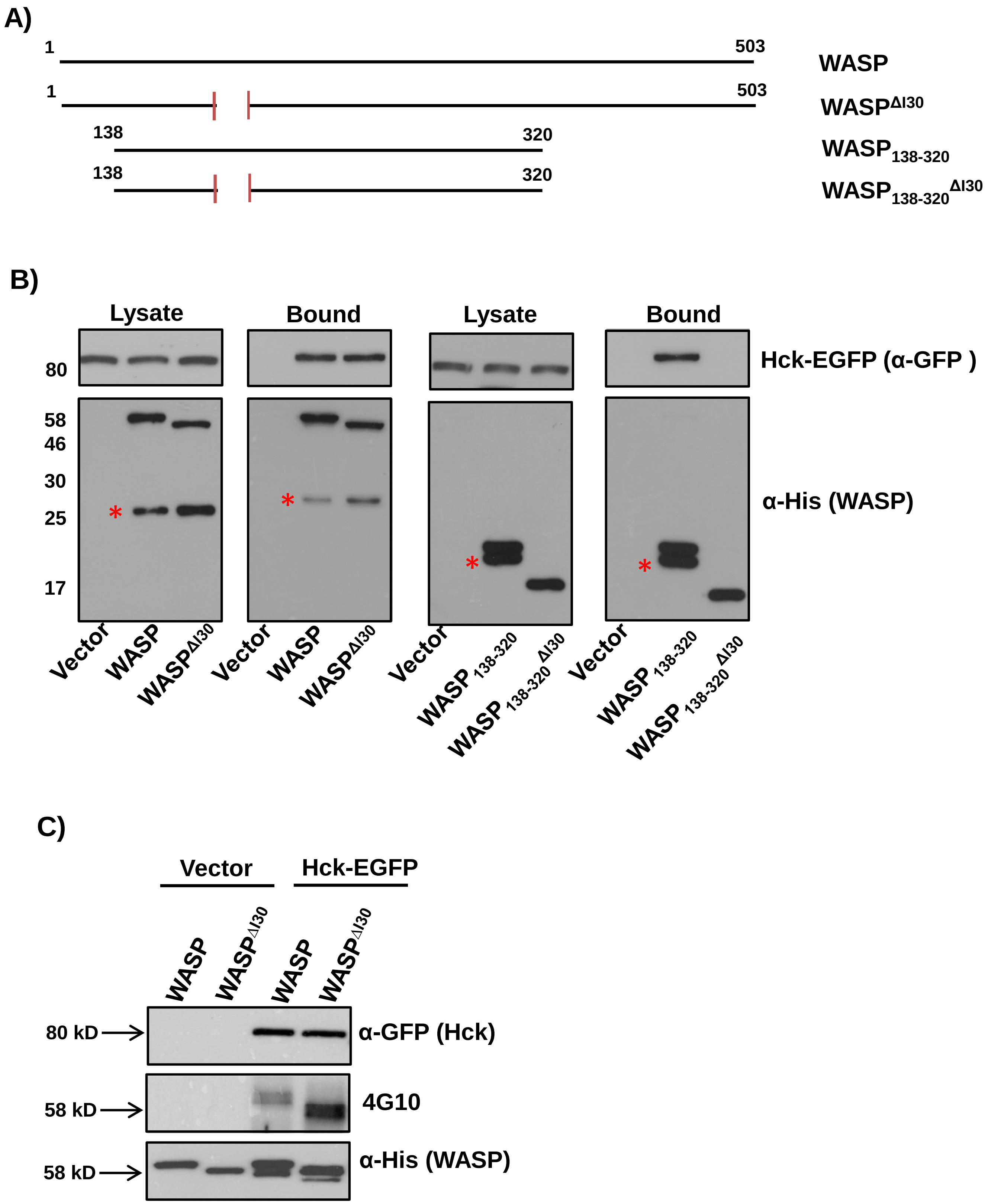
A)
1
503
WASP
1
503
WASPΔI30
138
320
WASP138-320
138
320
WASP138-320ΔI30
B)
Lysate
Bound
Lysate
Bound
80
58
46
30
25
17
Hck-EGFP (α-GFP)
α-His (WASP)
Vector
WASP
WASPΔI30
Vector
WASP
WASPΔI30
Vector
WASP 138-320
WASP 138-320 ΔI30
Vector
WASP 138-320
WASP 138-320 ΔI30
C)
Vector
Hck-EGFP
WASP
WASPΔI30
WASP
WASPΔI30
80 kD
α-GFP (Hck)
58 kD
4G10
58 kD
α-His (WASP)

S6)

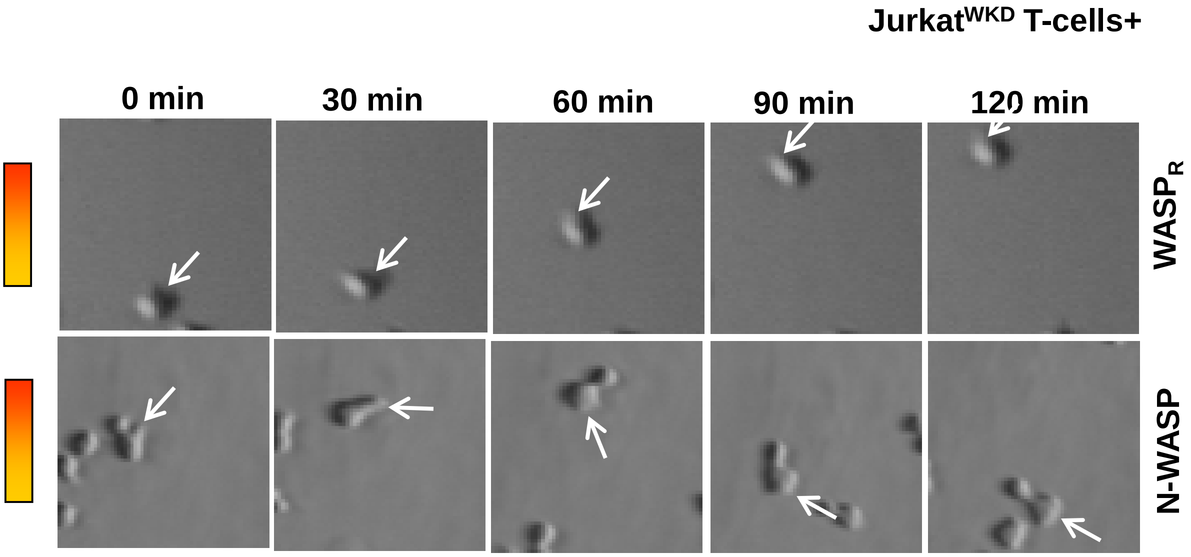
Jurkat^WKD T-cells+
0 min
30 min
60 min
90 min
120 min
WASP_R
N-WASP
